# Development of a three‐dimensional scintillation detector for pencil beam verification in proton therapy patient‐specific quality assurance

**DOI:** 10.1002/mp.17388

**Published:** 2024-09-10

**Authors:** Anne‐Marie Frelin, Gautier Daviau, My Hoang Hoa Bui, Cathy Fontbonne, Jean‐Marc Fontbonne, Dorothée Lebhertz, Erwan Mainguy, Cyril Moignier, Juliette Thariat, Anthony Vela

**Affiliations:** ^1^ Grand accélérateur National dʼIons Lourds (GANIL), CEA/DRF‐CNRS/IN2P3 Caen France; ^2^ Normandie University, UNICAEN Caen France; ^3^ Université de Caen Normandie ENSICAEN CNRS/IN2P3 Caen France; ^4^ Medical Physics Department CLCC François Baclesse Caen France

**Keywords:** 3D scintillator, patient‐specific quality assurance, proton therapy pencil beam scanning

## Abstract

**Background:**

Pencil Beam Scanning proton therapy has many advantages from a therapeutic point of view, but raises technical constraints in terms of treatment verification. The treatment relies on a large number of planned pencil beams (PB) (up to thousands), whose delivery is divided in several low‐intensity pulses delivered a high frequency (1 kHz in this study).

**Purpose:**

The purpose of this study was to develop a three‐dimensional quality assurance system allowing to verify all the PBs’ characteristics (position, energy, intensity in terms of delivered monitor unit—MU) of patient treatment plans on a pulse‐by‐pulse or a PB‐by‐PB basis.

**Methods:**

A system named SCICOPRO has been developed. It is based on a 10 × 10 × 10 cm^3^ scintillator cube and a fast camera, synchronized with beam delivery, recording two views (direct and using a mirror) of the scintillation distribution generated by the pulses. A specific calibration and analysis process allowed to extract the characteristics of all the pulses delivered during the treatment, and consequently of all the PBs. The system uncertainties, defined here as average value + standard deviation, were characterized with a customized irradiation plan at different PB intensities (0.02, 0.1, and 1 MU) and with two patient's treatment plans of three beams each. The system's ability to detect potential treatment delivery problems, such as positioning errors of the treatment table in this work (1° rotations and a 2 mm translation), was assessed by calculating the confidence intervals (CI) for the different characteristics and evaluating the proportion of PBs within these intervals.

**Results:**

The performances of SCICOPRO were evaluated on a pulse‐by‐pulse basis. They showed a very good signal‐to‐noise ratio for all the pulse intensities (between 2 × 10^−3^ MU and 150 × 10^−3^ MU) allowing uncertainties smaller than 580 µm for the position, 180 keV for the energy and 3% for the intensity on patients treatment plans. The position and energy uncertainties were found to be little dependent from the pulse intensities whereas the intensity uncertainty depends on the pulses number and intensity distribution. Finally, treatment plans evaluations showed that 98% of the PBs were within the CIs with a nominal positioning against 83% or less with the table positioning errors, thus proving the ability of SCICOPRO to detect this kind of errors.

**Conclusion:**

The high acquisition rate and the very high sensitivity of the system developed in this work allowed to record pulses of intensities as low as 2 × 10^−3^ MU. SCICOPRO was thus able to measure all the characteristics of the spots of a treatment (position, energy, intensity) in a single measurement, making it possible to verify their compliance with the treatment plan. SCICOPRO thus proved to be a fast and accurate tool that would be useful for patient‐specific quality assurance (PSQA) on a pulse‐by‐pulse or PB‐by‐PB verification basis.

## 1 INTRODUCTION

The use of proton beams for radiotherapy has advantageous ballistic properties compared to photon radiation therapy as charged particles have a limited range allowing for better sparing of normal tissue in many indications.^1^, ^2^, ^3^, ^4^, ^5^ In this therapeutic field, pencil beam scanning (PBS) also presents advantages over passive scattering proton therapy, with easier conformation of the dose to the target volume, not requiring individual collimator and bolus, and less secondary neutrons production. Nevertheless, this technique delivers the treatment through numerous pencil beams (PBs), up to several thousand.

Additionally, in the case of PBS performed with the IBA Proteus®ONE system, the IBA proprietary blind golfer algorithm (BGA) strategy is applied to minimize dose delivery uncertainties related to the cyclotron source. This strategy consists in dividing the planned pencil beams (PBs) delivery into several not consecutive pulses controlled by an internal feedback loop.^2^, ^3^ This raises difficulty to perform quality assurance (QA) of the treatments on a PB‐by‐PB basis, in a domain where the dosimetry tools are less numerous than in photon radiotherapy.

Most QA devices used in proton therapy are based on 1‐dimension (1D) or 2‐dimension (2D) arrays of ionization chambers, such as Zebra or MatriXX devices (IBA dosimetry, Schwarzenbruck, Germany). Even if these tools are robust and accurate, some of them suffer from a spatial resolution of several millimeters and don't allow PB‐by‐PB measurements. They also usually provide limited information on the PBs (for example depth dose distribution for the zebra) and performing three‐dimensional (3D) verifications can become extremely time‐consuming because it is necessary to reproduce the irradiation at different depths.

To overcome these limitations, scintillation detectors are very promising as they can provide a complete characterization of the delivered pulses and PBs. Scintillation dosimetry has been extensively studied in photon radiation therapy because of its advantageous properties, water‐equivalence, spatial resolution, real‐time measurement, shape versatility,^4^, ^5^, ^6^, ^7^, ^8^, ^9^ and has overcome the main drawback of the production of parasitic Čerenkov light.^7^, ^10^, ^11^ With the progress in image acquisition and processing, studies have shown the highly desirable possibility to perform 3D dosimetry from volumetric scintillation materials.^12^, ^13^, ^14^

The studies on scintillating systems are more scarce in proton therapy, partly due to the more recent democratization of proton therapy and consequently of associated dosimetry tools. Early experiments were carried out with plane scintillators to measure 2D dose distributions^15^ and currently the Lynx system (IBA dosimetry, Schwarzenbruck, Germany) is commercially available for 2D relative dose profile measurements and PBS daily QA. Volumetric scintillation dosimetry was also studied with liquid scintillators and plastic scintillators, showing good performance to verify position and range of proton spots and measure depth dose profiles.^16^, ^17^, ^18^, ^19^

The progress in camera acquisition speed is a real opportunity of improvement in this domain by allowing dosimetry on a pulse‐by‐pulse or PB‐by‐PB basis. Recent works were carried out in this direction, to measure proton range and SOBP width^20^ and 2D dose and dose rate maps.^21^, ^22^

Scintillation dosimeters could be particularly interesting in the domain of patient‐specific QA (PSQA) which is very time‐consuming and involves in‐phantom 2D measurements. These measurements are performed for a limited number of planes and a limited spatial resolution. To reduce the time spent on these measurements, the question of applying measurement‐less methods thus arises and various studies consider the possibility to perform PSQA from an analysis of the machine log‐files recorded during irradiation coupled to MC simulations.^23^, ^24^, ^25^ This approach presents the interest of not necessitating additional QA measurements and to allow verifications on the treatment actually delivered to the patient. On the other hand, it doesn't constitute an independent verification of the irradiation system, provide no information on energy variations and doesn't allow to detect errors or deviations from external origins. Scintillation dosimeters, thus could be used to perform more complete PBs delivery verifications as well as high‐resolution 3D dosimetry for end‐to‐end QA.

In this study, we focused on the PBs verification and we developed a new 3D scintillation system, based on an ultra‐fast CMOS camera, able to record each pulse of a treatment plan, at a frequency of 1 kHz. This system was used to perform a fast and accurate PB‐by‐PB QA of patient's treatments in a single irradiation. The performances of the system were firstly characterized on a pulse‐by‐pulse basis. The agreement between planned and delivered PBs characteristics (position, energy and intensity in monitor units—MU) were then verified for several treatment plans. Finally, the ability of the system to detect positioning errors was evaluated.

## 2 MATERIALS AND METHODS

### 2.1 Pencil beam scanning irradiations with the IBA Proteus^®^ONE

This study was performed in PBS with the IBA Proteus®ONE at Cyclhad Proton therapy center (Caen, France). PBS consists in irradiating the target volume with a finite number of PBs distributed in a finite number of energy layers covering the depth of the target volume. The number of PBs necessary to achieve the prescribed dose distribution (up to thousands) as well as their characteristics (position in the isocenter reference frame, energy, intensity in MU) are generally planned by the treatment planning system (TPS). Customized irradiations can also be manually defined in a PBS layer definition text file (PLD file).

The dosimetry system developed in this study was designed to measure the characteristics of the PBs delivered by the IBA Proteus®ONE system for the treatment of small volume and to verify their compliance with the treatment plan, in the context of PSQA. This system has specific constraints: a high beam delivery frequency (1 kHz) and the use of the BGA delivery strategy. Consequently, each energy layer is scanned several times (three−four scans per layer in this study) and inside each layer scan, each PB can be delivered in several pulses (from 3 to 11 pulses per PB in this study).^2^, ^3^

Both these specificities are challenging for PB verification, and it must be noted that the BGA significantly complicates the verification of the delivered PBs characteristics as each PB is delivered in several pulses not all consecutive.

### 2.2 Experimental setup

**FIGURE 1:**
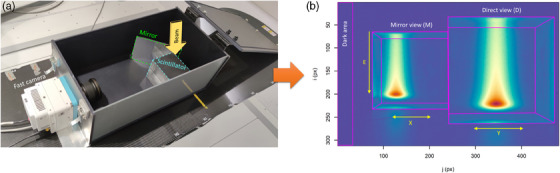
(A) Experimental setup on the treatment table. It is composed of a 10 × 10 × 10 cm^3^ BC‐412 plastic scintillator, a mirror oriented at 45° relative to the cube and a fast camera. (B) Typical image acquired when the cube is irradiated by vertical proton beams. The camera records a direct view of the pulse on the right side of the image (D) and a view of the pulse reflected by the mirror on the left side of the image (M). The background signal is calculated in a dark area on the left side of the sensor and subtracted sensor line by sensor.

The device developed in this study, called SCICOPRO and represented in Figure 1 (left), was designed to perform verifications on patient treatment plans using small irradiation fields (<6 × 6 cm^2^). It is based on the recording by a fast camera of the scintillation light emitted by a 10 × 10 × 10 cm^3^ phantom of BC‐412 plastic scintillator (Saint‐Gobain Crystals, Nemours, France) placed at about 32 cm from the camera sensor. This scintillator has a mass density of 1.023 g cm^−3^ close to water density and an emission spectrum centered on 430 µm. Four faces were painted black to limit optical reflections.

A first‐surface mirror of 13 × 18 cm^2^ was positioned at the left of the scintillator at an angle of 45°. In this application, the scintillator must be irradiated by vertical beams. The camera then records a direct view (D) of about 230 × 230 pixels of the pulse on the right side of the image (apparent pixel size of about 435 µm for the scintillator face closest to the camera) and a view of about 167 × 167 pixels of the pulse reflected by the mirror (M) on the left side of the image (apparent pixel size of about 600 µm for the scintillator face closest to the camera) (see Figure 1 (right)). The horizontal position of the pulse in the direct and mirror views are directly related to the *Y* and *X* positions respectively in the isocenter reference frame. The range of the pulse in the phantom is related to its energy and the maximum pixel value is related to the pulse intensity. The setup configuration and the mirror thus allow the determination of all the PBs characteristics in a single acquisition.

A light‐shielding box prevents the measurement of parasitic ambient light and has a 5‐mm thick beam entry window made of polyvinyl chloride (PVC), adapted to vertical beam irradiations. The center of the scintillator cube was placed at the irradiation isocenter.

Given the high delivery frequency of the proteus®ONE, the CMOS ultra‐fast Phantom VEO‐E‐1310L camera (Vision Research Inc, Phantom Cameras, Wayne, USA), adapted to cinematic applications, was chosen. Its fast acquisition speed (up to 10,860 images per second) and its sensitivity allow the acquisition of each pulse delivered by the proteus®ONE at the frequency of 1 kHz during the irradiation. The acquisition was synchronized to a logic signal delivered by the Proteus®ONE with an exposure time of 990 µs ensuring to record the scintillation signal without delay adjustment.

### 2.3 Image analysis

In this work, the image analysis mainly consists in determining pulses position and intensity. But it first requires to sort and preprocess raw pulse images. The different steps of these are detailed in this section.

#### 2.3.1 Image preprocessing

The logic signals provided by the Proteus®ONE points out the extraction of the beam from the cyclotron instead of the beam presence in the treatment room. Consequently, it triggers the acquisition of a significant number of dark images by the camera in addition to pulse images (between 10% and 60% in this study). The first step of the preprocessing was thus to sort dark and pulse images. To that intent, pulse images were identified by the presence of clusters of more than 50 adjacent pixels presenting an intensity higher than the standard deviation of dark images.

Hot pixels caused by scattered radiations directly hitting the camera sensor were then identified from their intensity and their spatial gradient and corrected from neighboring pixels’ value. The average dark image was subtracted from pulse images. Additionally, temporal variations of the background were corrected image by image, by subtracting the average value of the leftmost 30 pixels of the image calculated for each sensor line (noted “Dark area” in Figure 1B).

#### 2.3.2 Pulse images analysis

**FIGURE 2:**
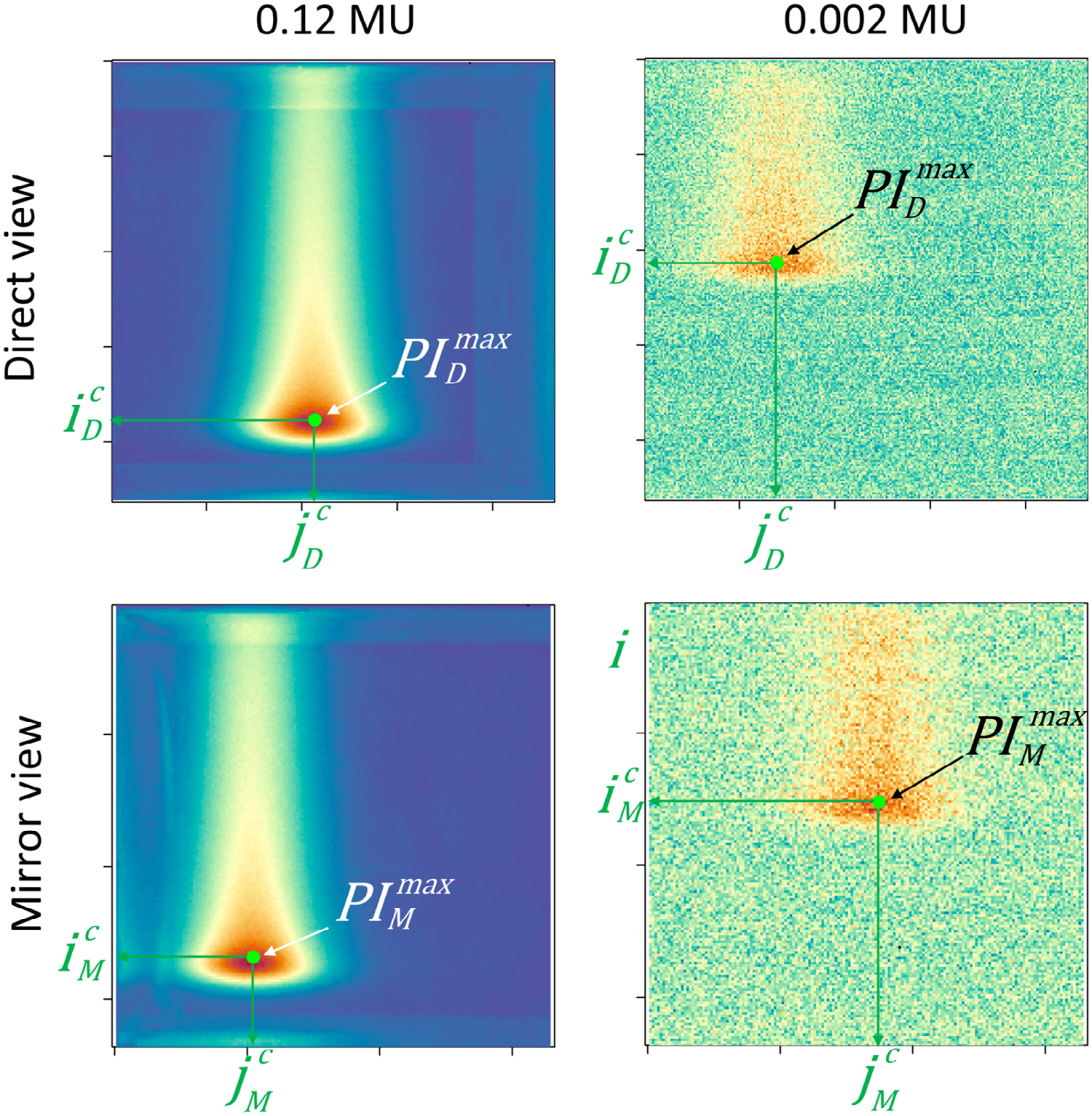
Direct and mirror views of a high intensity pulse (0.12 MU) on the left side and a low intensity pulse (0.002 MU) on the right side. The scintillation distributions are characterized by the position and the intensity of the Bragg peak, determined by Maximum likelihood estimation and noted by the green point in each view.

As this study primarily focused on the verification of PB characteristics (position, energy, and intensity in MU), the analysis of images focused on the determination of these quantities in the pulse images. As described in Section 2.2, two different views of the scintillation distribution were extracted from the image: the direct view and the mirror view, shown in Figure 2 for a high (on the left side) and a low pulse intensity (on the right side), respectively 0.12 and 0.002 MU.

The scintillation distribution was characterized in each view (D and M), by the position $\left(i_{X}^{c},j_{X}^{c}\right)$ and the maximum intensity $PI_{X}^{max}$ of the Bragg peak, with X=DorM. These quantities were determined by Maximum likelihood estimation, the likelihood estimator being a 2‐dimensional Gaussian distribution of six pixels standard deviation:

(1) $$L_{X}\left(i,j\right)=\sum_{\left(k,l\right)\;in\;image}Gauss\left(i,j,k,l\right)\times view_{X}\left(k,l\right)$$

with X=DorM, (k,l) the pixel coordinates and Gauss(i,j,k,l) the value of the Gaussian distribution centered on (i,j) at the pixel position (k,l).

Consequently, $\left(i_{D}^{c},j_{D}^{c}\right)$ and $\left(i_{M}^{c},j_{M}^{c}\right)$ are the parameters maximizing $L_{D}$ and $L_{M}$ respectively, and $L_{X}\left(i_{X}^{c},j_{X}^{c}\right)$ is a proportional estimator of the maximum intensity $PI_{X}^{max}$. The positions found by this method in both views are indicated by the green dots in Figure 2 for the two intensities. They show that even at very low intensity (0.002 MU on the right side of Figure 2, corresponding to a maximum dose of approximately 10^−4^ Gy at the Bragg peak position), the signal‐to‐noise ratio is sufficient to determine the scintillation distribution characteristics.

It can be noted that $i_{M}^{c}$ and $PI_{M}^{max}$ are redundant with $i_{D}^{c}$ and $PI_{D}^{max}$ and their determination is not necessary. $PI_{D}^{max}$ and $i_{D}^{c}$ were then chosen because of the slightly higher scintillation intensity and better signal‐to‐noise ratio obtained in the direct view.

### 2.4 Calibration

**FIGURE 3:**
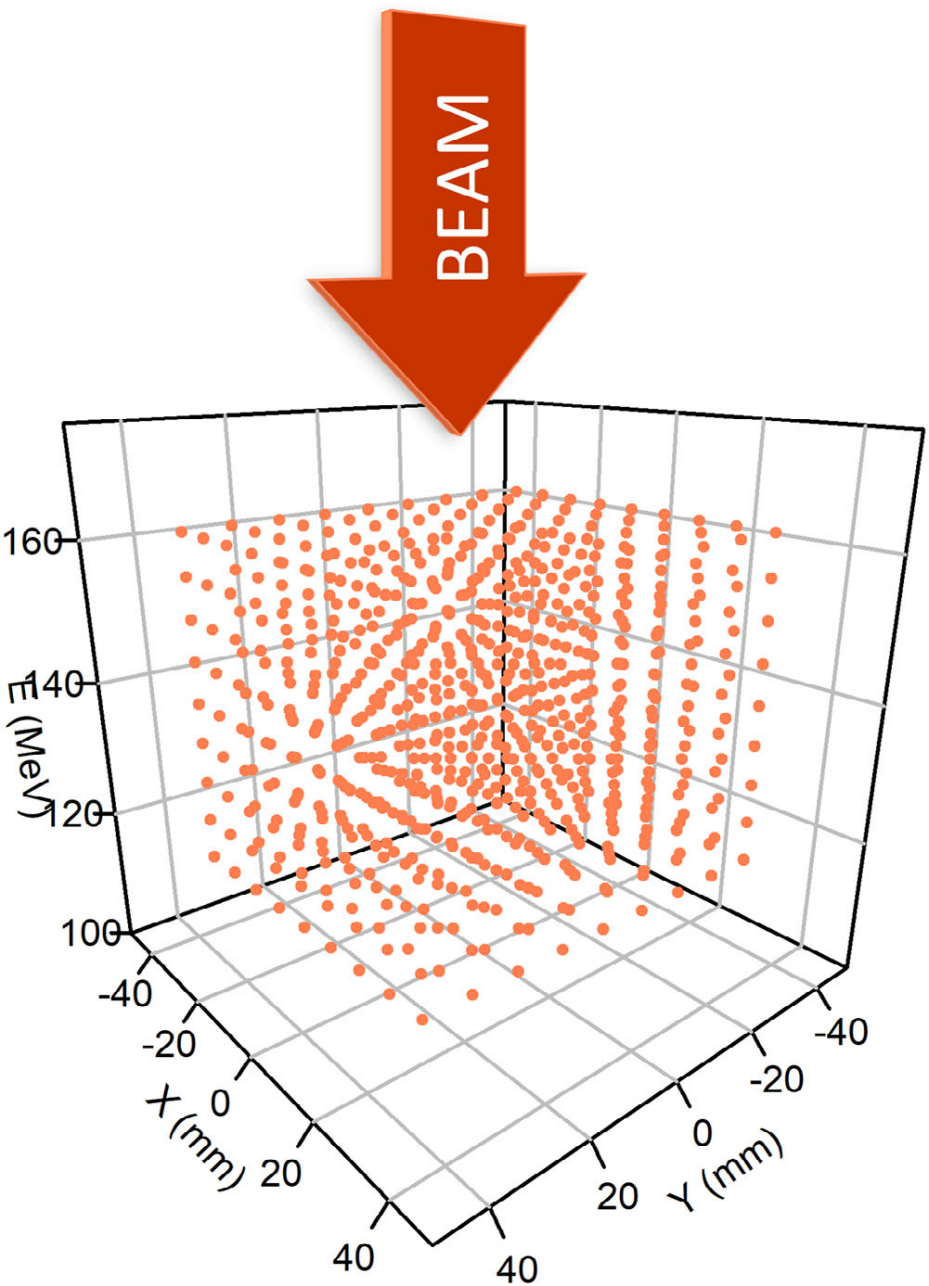
Calibration irradiation plan. The scintillator cube (represented in solid black lines is irradiated by 729 PBs, whose positions are represented by the color dots, distributed into nine beam energy layers. Each layer includes 9 × 9 PBs whose position is uniformly distributed between −40 and +40 mm in both *X* and *Y* directions. The calibration irradiation was performed twice: once at 1 MU and once at 0.1 MU.

In a second step, the pulses characteristics of the scintillation distributions $\left(i_{D}^{c},j_{D}^{c},j_{M}^{c},\;PI_{D}^{max}\right)$ measured in the image were converted into pulse characteristics (positions (X,Y), energy E and intensity I in MU) thanks to a specific calibration irradiation plan illustrated in Figure 3. This irradiation plan was composed of 729 PBs divided into nine energy layers between 120.8 and 161.8 MeV. A range shifter of 7.4 cm water‐equivalent thickness was used to match the scintillator dimensions. In each energy layer, the positions of 81 PBs were uniformly distributed between −40 and +40 mm in the *X* and *Y* directions. The irradiation plan was repeated twice. Once with PB intensities of 1 MU and once with PB intensities of 0.1 MU to cover all the range of pulses’ intensities (see Section 3.1).

It must be noted that the PBs delivery in several pulses has a significant impact on the measurement of a PB intensity, which actually corresponds to the measurement of several lower intensities. Consequently, in this work the calibration was performed between pulse images and pulse characteristics provided by the machine log‐files recorded during irradiation.^26^, ^27^, ^28^ It is indeed the only way to get the characteristics (in particular the intensity) on a pulse‐by‐pulse basis. This approach also allowed to study the effect of the BGA on SCICOPRO measurements.

The log‐file pulse positions and intensity are measured by a stripped ionization chamber at the nozzle level. Consequently, the log‐file positions were converted to the (*X*,*Y*) positions in the patient/isocenter reference frame, taking into account the geometric transformations but also the beam deflection.

After this conversion, the log‐files pulse data were associated to the corresponding pulse images recorded by the camera by temporal synchronization of the log‐files entries and images timestamps. A calibration function was then established between the pulses characteristic $P_{burst}$ (with P=X,YorE) and $I_{burst}$ provided by the log‐files and the scintillation distribution characteristics $\left(i_{D}^{c},j_{D}^{c},j_{M}^{c},\;PI_{D}^{max}\right)$:

(2) $$\left\{\begin{array}{c} P_{burst}=f\left(i_{D}^{c},j_{D}^{c},j_{M}^{c}\right)\\ I_{burst}=g\left(i_{D}^{c},j_{D}^{c},j_{M}^{c},\;PI_{D}^{max}\right) \end{array}\right.$$

with f a linear combination of polynomial functions (powers up to 5) of $i_{F}^{c},\;j_{F}^{c}$ and $j_{L}^{c}$, and g a linear combination of polynomial functions (powers up to 4) of $i_{F}^{c},\;j_{F}^{c},\;j_{L}^{c}$ and $PI_{D}^{max}$.

In order to avoid overfitting, a model deletion approach by minimization of Bayesian Information Criterion (BIC) was used. The model achieving the lowest BIC was selected, giving less than 118 over 215 possible parameters for f and 230 over 624 possible parameters for g.

This calibration directly takes into account and corrects the perspective effect, optical artifacts (diffraction, vignetting, light attenuation…), scintillation quenching and the camera response (uniformity and linearity).

### 2.5 Pencil beams reconstruction

Finally, the PBs’ intensity $I_{PB}$ was calculated as the sum of the intensities of the n pulses arriving at the same position, and the other PB features $P_{PB}$ (position and energy) as the average feature of the n pulses, weighted by their intensities:

(3) $$\left\{\begin{array}{c} I_{PB}=\sum_{i=1}^{n}I_{burst}^{i}\\ P_{PB}=\frac{\sum_{i=1}^{n}I_{burst}^{i}\times P_{burst}^{i}}{I_{PB}} \end{array}\right.$$

with $I_{burst}^{i}$ and $P_{burst}^{i}$ the intensity and other features of the pulse i∈[1;n] corresponding to a given PB.

In this work, the pulses corresponding to a same PB were regrouped thanks to a PB identification number provided by the log‐file.

### 2.6 Evaluation of SCICOPRO performance

The main goal of this study being to verify PBs’ characteristics, the performances of our system were characterized in terms of position (X,Y), energy and intensity precision and accuracy.

The evaluation was performed with a customized irradiation grid, similar to the calibration irradiation represented Figure 3 but irradiating a smaller volume. It was composed of 729 PBs, with energies distributed between 120 and 156 MeV and positions distributed between −30 and 30 mm in the *X* and *Y* distributions. This spatial range is representative of the target volume addressed in this study (6 × 6 × 6 cm^3^). This irradiation grid was tested at different PB intensities: 0.02, 0.1, and 1 MU.

Given the BGA delivery of the Proteus®ONE, the performances of SCICOPRO were first evaluated on individual pulses by comparison with the log‐files data:

(4) $$d_{P}^{i}=P_{log}^{i}-P_{meas}^{i}$$

with $P_{log}^{i}$ and $P_{meas}^{i}$ the characteristics provided by the log‐file and measured with SCICOPRO for each pulse of index i, and Pin(X,Y,E,I).

As stated earlier, the evaluation on a pulse‐by‐pulse basis is necessary to evaluate the impact of the BGA delivery on the measurements.

### 2.7 Verification of planned PB characteristics

In a second step, SCICOPRO was tested to measure and check PBs’ characteristics of patient treatment plans.

A Computed Tomography (CT) scan of SCICOPRO was performed beforehand, with the scintillator cube center at isocenter and the Water Equivalent Thickness (WET) of the entry window accurately measured with the Zebra system (IBA dosimetry, Schwarzenbruck, Germany) and integrated to the treatment plan. Two treatments plans (named “P1” and “P2”) of three beams each (named “B1”, “B2” and “B3”) were then planned on our setup by the treatment planning system (TPS). All treatment plans and irradiations were performed with vertical beams and the center of the scintillator positioned at the isocenter.

Contrary to the previous section, the discrepancies were calculated here between measured and planned PB characteristics provided by the TPS:

(5) $$d_{P}^{i}=P_{RTplan}^{i}-P_{meas}^{i}$$

with $P_{RTplan}^{i}$ and $P_{meas}^{i}$ the PBs’ characteristics provided by the RT Plan and measured with SCICOPRO respectively for each PB of index i, and Pin(X,Y,E,I).

The distribution of these differences were used to calculate 99.7% confidence intervals ($CI_{P}$) for each characteristic:

(6) $$CI_{P}=\left[\bar{d_{P}}-3\times sd\left(d_{P}\right);\bar{d_{P}}+3\times sd\left(d_{P}\right)\right]$$

with Pin(X,Y,E,I). The CIs are redundant with the mean value and the standard deviation of the differences, but they can be used as criterions to evaluate the proportion of PBs within these CIs as it will be exposed in the next section.

### 2.8 Detection of positioning errors of the treatment table

The detection of positioning errors with SCICOPRO was performed by assessing the proportion of PBs within the confidence intervals defined in the previous section. These intervals are directly representative of SCICOPRO performances and allow to evaluate the detection of errors close to the limits of the system.

For this evaluation, an irradiation was planned with the TPS to deliver a homogeneous dose distribution of 0.8 Gy in a sphere of 5 cm‐diameter placed at the center of the phantom scintillator. The irradiation was delivered four times: one in nominal irradiation conditions, two with a slight rotation of the treatment table (1° rotation and 1° pitch) and one with a 2‐mm translation. For each irradiation the proportion of PBs within the CIs was evaluated.

## 3 RESULTS

### 3.1 Performance of SCICOPRO

**FIGURE 4:**
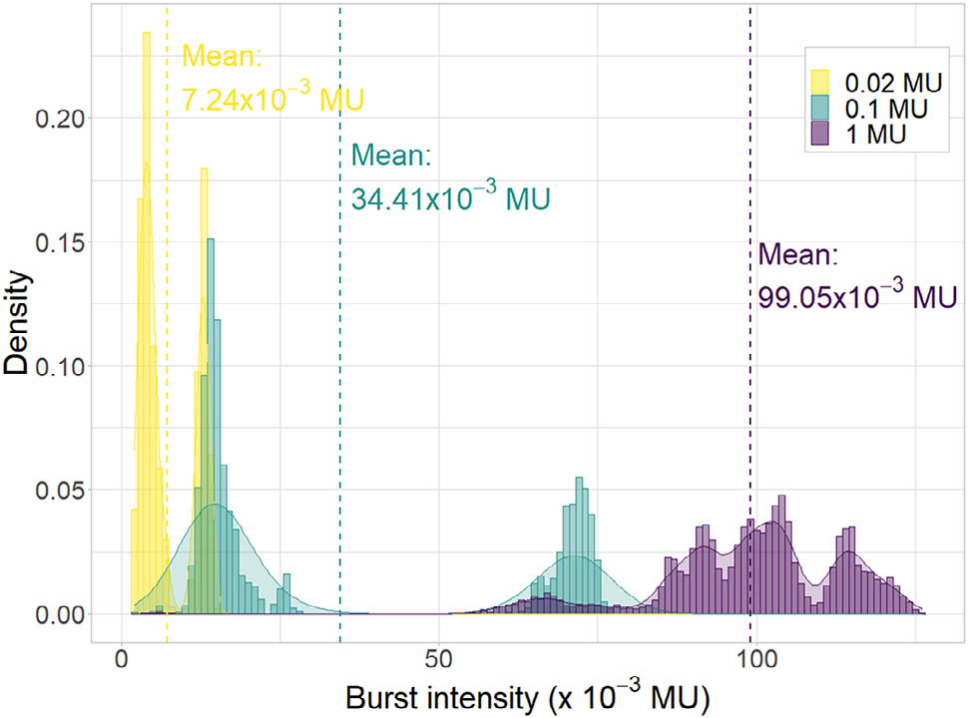
Probability density histogram (in bars) and the corresponding smoothed function (in solid line) of the pulses’ intensity delivered for the grid irradiation at 0.02, 0.1, and 1 MU (resp. in yellow, cyan and purple). The mean intensity values are indicated by the dashed vertical lines.

The first step of this study was to evaluate SCICOPRO performance in terms of spatial, energy and intensity resolution at different intensity levels. Consequently, irradiations were performed at 0.02, 0.1, and 1 MU. As shown in Figure 4, the intensity of the pulses delivered by the Proteus®ONE stayed in a limited range with a maximum intensity of about 0.13 MU whatever the planned PB intensity, but variable distributions in this range. The delivery of the planned intensity is achieved by increasing the number of delivered pulses per PB rather than increasing the pulses intensity: up to four pulses per PB at 0.02 and 0.1 MU and up to 11 pulses per PB at 1 MU. The same initial number of 729 PBs thus leads to the delivery of 2018 pulses at 0.02 MU, 2127 pulses at 0.1 MU and 7380 pulses at 1 MU.

The mean value and the standard deviation of the discrepancies $d_{P}^{i}$ calculated between the pulse characteristics provided by the log‐files and measured by SCICOPRO for the pulses are summarized in Table 1. They show a slight decrease of the standard deviations with the planned intensity, explained by the higher pulse's intensities shown by Figure 4 (7.24 × 10^−3^ MU, 34.41 × 10^−3^ MU and 99.05 × 10^−3^ MU at 0.02, 0.1, and 1 MU, respectively). Nevertheless, this variation remains limited due to the limited range of the pulse intensities (below about 0.13 MU) whatever the planned intensity and the good signal to noise ratio, even for low‐intensity pulses, showed by Figure 2.

**TABLE 1:** Mean value ± standard deviation of the discrepancies between the characteristics provided by the log‐files and measured by SCICOPRO, calculated on a pulse‐by‐pulse basis and a PB‐by‐PB basis, at planned intensities of 0.02, 0.1, and 1 MU per PB.

	RUN	0.02 MU	0.1 MU	1 MU
	Total nb of bursts	2018	2127	7380
Burst by burst	$\bar{d_{X}}\pm sd\left(d_{X}\right)$(µm)	88 ± 392	123 ± 344	112 ± 332
	$\bar{d_{Y}}\pm sd\left(d_{Y}\right)$(µm)	36 ± 350	24 ± 264	20 ± 248
	$\bar{d_{E}}\pm sd\left(d_{E}\right)$(keV)	−16 ± 184	−2 ± 173	31 ± 175
	$\bar{d_{I}}\pm sd\left(d_{I}\right)$ (10^‐3^ MU)	0.23 ± 0.48	0.38 ± 0.50	0.37 ± 0.63
PB by PB	$\bar{d_{X}}\pm sd\left(d_{X}\right)$(µm)	89 ± 297	113 ± 302	121 ± 277
	$\bar{d_{Y}}\pm sd\left(d_{Y}\right)$(µm)	37 ± 247	26 ± 215	23 ± 193
	$\bar{d_{E}}\pm sd\left(d_{E}\right)$(keV)	−29 ± 146	13 ± 146	34 ± 143
	$\bar{d_{I}}\pm sd\left(d_{I}\right)$(10^‐3^ MU)	0.64 ± 1.15	1.10 ± 0.99	3.80 ± 5.07

These results are translated in even closer standard deviations for the discrepancies calculated PB by PB and summarized in the lower part of Table 1. Indeed, the slight advantage given by higher pulse intensities is counterbalanced by the higher number of delivered pulses and consequently by the addition of errors in the PB reconstruction. In particular, the absolute uncertainty on the PB intensity is the highest at 1 MU with a standard deviation of more than 5 × 10^−3^ MU (which nevertheless corresponds to a relative uncertainty of 0.5%) and the addition of systematic errors.

These performances are very promising with no significant bias and standard deviations of the order of 300 µm for position measurement, and 150 keV for energy measurements on a PB‐by‐PB basis. These dispersions include a standard deviation of about 35 µm on the positions given by the log‐files for the pulses corresponding to the same PB. The uncertainty on the intensity measurement is the most sensitive to the pulse intensity distribution. Although very low, the discrepancies have the same order of magnitude as the lowest pulse intensities (of the order of 10^−3^ MU), and thus will be correlated to the pulses’ intensity distribution.

### 3.2 Verification of planned PB characteristics

**FIGURE 5:**
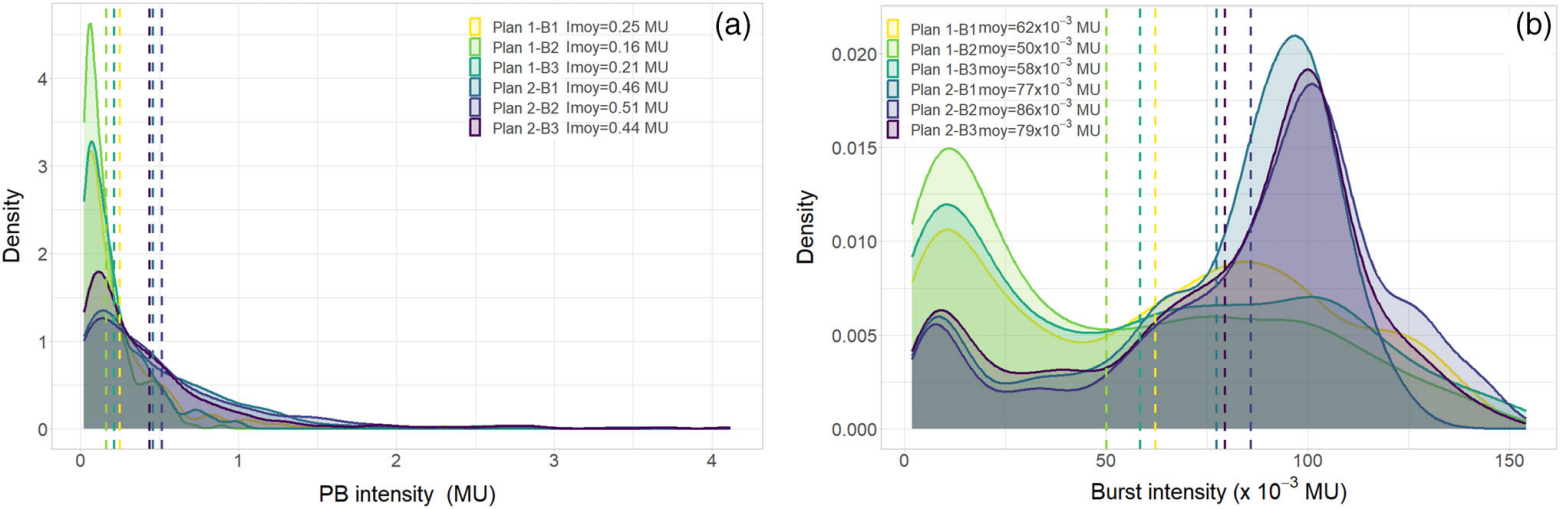
(A) Probability density of the planned PBs’ intensity. (B) Probability density of the intensities of the pulses delivered for each irradiation. The mean intensity values are indicated by the dashed vertical lines.

As shown in the performance evaluation of the previous section, the precision of the PBs’ intensity measurements mainly depends on pulses’ intensity distribution. To evaluate this effect and the performance of SCICOPRO in realistic conditions, six patient clinical irradiation fields planned by the TPS were evaluated. Figure 5 shows the distributions of the planned PBs’ intensities (A) and the pulses’ intensities (B) for the different irradiations. Even if the different irradiation plans show different PBs’ intensity distributions, with intensities reaching 4 MU, these intensities are actually divided into pulse whose intensities stay in a range comparable with the one of Section 3.1. Nevertheless, it can also be noticed that the six irradiations have significantly different distributions.

**FIGURE 6:**
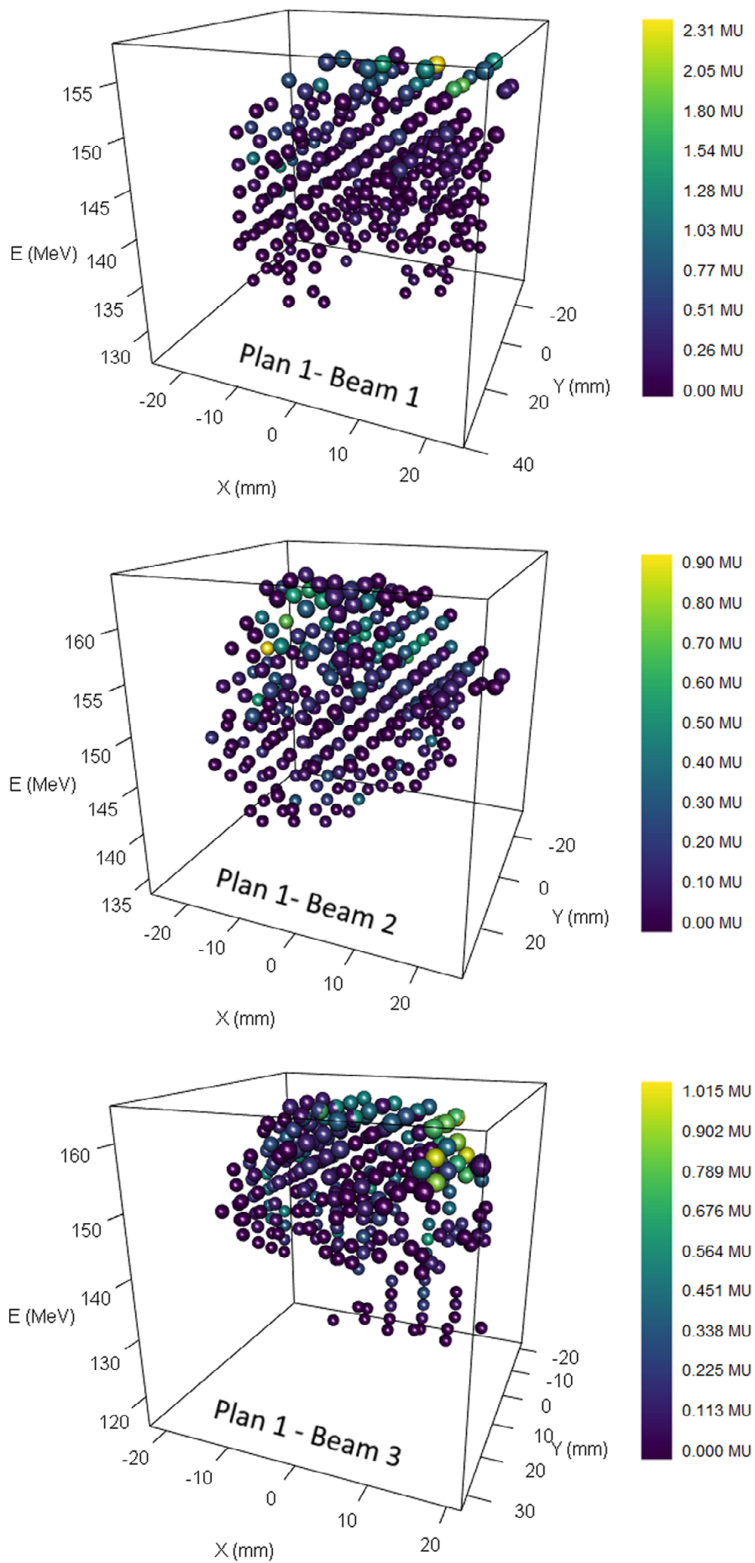
PBs characteristics (position, energy and intensity in color scale) measured with SCICOPRO for the treatment plan 1.

One example of the treatment plan PBs reconstruction is shown in Figure 6. It corresponds to the three beams of the 1st irradiation plan. For clarity, only the measured PBs characteristics are displayed, but they match the planned characteristics.

The mean value and the standard deviations of the discrepancies between measured and planned characteristics are summarized in Table 2. The results given in this table are consistent with the performances measured in the previous section. Even if a slight systematic error is observed on the X position measurements, the dispersion is very low and well under 1 mm. It should be noted that these uncertainties include possible delivery errors and the log‐file positions uncertainties resulting from the calibration process. A study of machine log‐files uncertainties reported that PBs were delivered with a position accuracy better than 200 µm and that the positions recorded in the log‐file suffered from systematic uncertainties depending on the PB position and random uncertainties below 200 µm.^28^ In this study, the positions discrepancies (mean value ± standard deviation calculated over the six irradiation) between the positions of the log‐files and the PLD positions (thus including the delivery and the log‐file uncertainties) were (63±147)μm in the X direction and (3±116)μm in the Y direction, which is not negligible compared to the discrepancies measured with SCICOPRO. The standard deviations for the measurement of the PBs’ intensity (between about 1.6 and 5 × 10^−3^ MU) corresponds to the upper values measured in the performance evaluation. But they also are consistent with the mean values of the pulses intensities (between 50 and 86 × 10^−3^ MU). Nevertheless, they correspond to relative dispersions smaller than 2.6%. It can also be noted that the PB‐by‐PB discrepancies lead to relative differences smaller than 1.06% on the total amount of MUs delivered during the irradiation for the six tested irradiation beams.

**TABLE 2:** Mean value ± standard deviation of the discrepancies between the planned characteristics of PBs provided by the TPS and the ones measured by SCICOPRO.

	P1‐B1	P1‐B2	P1‐B3	P2‐B1	P2‐B2	P2‐B3
nb of PBs/nb of bursts	285/1146	212/695	220/798	151/898	300/1800	248/1366
$I_{tot}$ (MU)	71.07	43.90	57.82	92.79	167.01	133.01
$\bar{d_{X}}\pm sd\left(d_{X}\right)$(µm)	162 ± 261	315 ± 213	41 ± 345	295 ± 281	219 ± 329	138 ± 266
$\bar{d_{Y}}\pm sd\left(d_{Y}\right)$(µm)	52 ± 212	16 ± 217	36 ± 194	−6 ± 194	45 ± 236	−25 ± 241
$\bar{d_{E}}\pm sd\left(d_{E}\right)$ (keV)	−4 ± 133	−49 ± 128	−54 ± 127	−31 ± 135	−48 ± 132	−63 ± 145
$\bar{d_{I}}\pm sd\left(d_{I}\right)$ (10^‐3^ MU)	0.80 ± 2.21	0.28 ± 1.59	0.47 ± 2.13	0.79 ± 4.45	−0.29 ± 4.72	−0.47 ± 4.72
$\bar{d_{I}}/I\pm sd\left(d_{I}/I\right)$ (%)	0.78 ± 1.84	0.41 ± 1.87	0.37 ± 2.56	0.25 ± 1.61	0.30 ± 2.09	0.01 ± 1.48
$\sum dI/I_{tot}$ (%)	0.32	0.12	0.24	1.06	0.47	−0.05

The distributions of the discrepancies determined on all the six irradiation fields were used to calculate 3‐σ confidence intervals (99.7% for normal distribution) that will be used in the next section to detect positioning errors: $CI_{X}=\left[-713;1090\right]\;\mu m$, $CI_{Y}=\left[-638;684\right]\;\mu m$, $CI_{E}=\left[-446;364\right]\;\mathrm{keV}$ and $CI_{I}=\left[-5.52;6.26\right]\%$.

### 3.3 Detection of positioning errors of the treatment table

Finally, the ability of SCICOPRO to detect treatment delivery problems, such as positioning errors of the treatment table in this work, was evaluated by calculating the proportion of PBs within the CIs determined in the previous section for a 5 cm diameter sphere irradiation. The irradiation was performed first under nominal conditions and then with three different positioning errors: two rotations and one translation of the treatment table. The results are summarized in Table 3.

**TABLE 3:** Proportion of PBs within the *X*, *Y*, *E*, and *I* confidence intervals and within the four intervals ($CI_{X}\cap CI_{Y}\cap CI_{E}\cap CI_{I}$) for the four irradiations.

	Nominal positioning	1° rotation	1° pitch	2 mm translation
in $CI_{X}=\left[-713;1090\right]\mu m$	99.70%	93.41%	87.72%	100%
in $CI_{Y}=\left[-638;684\right]\mu m$	99.70%	88.62%	96.41%	0%
in $CI_{E}=\left[-446;364\right]\;keV$	99.70%	99.40%	100%	100%
in $CI_{I}=\left[-5.52;6.12\right]\%$	99.10%	98.20%	98.20%	98.50%
in $CI_{X}\cap CI_{Y}\cap CI_{E}\cap CI_{I}$	98.20%	82.93%	83.23%	0%

In the case of the nominal conditions, more than 98% of the PBs are within the tolerance for all the PBs features (*X* and *Y* position, energy and intensity). On the other hand, in the case of the translation, the positioning error being much larger than the spatial performances of SCICOPRO, the proportion within all the CIs is logically null, and allow to clearly identify in which direction the error occurs. In the case of the rotations of the treatment table, even with very small angles, the proportion of PBs within the CIs significantly decrease, below 84%. These values prove the ability of SCICOPRO detect small positioning errors of the treatment table.

## 4 DISCUSSION

In this study an experimental device was developed to measure the characteristics of the PBs of patient treatment and verify their compliance with the treatment plan as part of PSQA in PBS. This device is adapted to the BGA delivery strategy which leads to the delivery of the planned PBs in several pulses of lower intensity. This strategy raises questions regarding the measurement of PBs characteristics as it actually consists in measuring the characteristics of several lower intensity images, thus inducing a multiplication of the noise and uncertainty sources.

To study this concern, the experimental setup was calibrated on a pulse‐by‐pulse basis from log‐files data, which is currently the only possibility. This method allowed to evaluate SCICOPRO performances but also the pulse intensity distribution in different irradiation configurations (customized irradiations with all the PBs at the same intensity and treatment plans provided by the TPS).

In terms of position and energy measurement, the study demonstrated the high sensitivity of SCICOPRO which proved able to measure proton pulses of intensities as low as 2 × 10^−3^ MU, corresponding to a maximum dose of about 10^−4^ Gy (as shown in Figure 2 left), leading to spatial and energy uncertainties almost independent from the intensity distribution. In the treatment verifications of Section 3.2, differences between planned and measured positions smaller than 580 µm (average value + standard deviation) were found. As specified in Section 3.2, this value includes uncertainties coming from the PB delivery and the log‐file data used for the calibration. This uncertainty is not negligible as difference as high as 210 µm (average value + standard deviation) have been calculated between planned and log‐file positions. It was reported elsewhere that delivery uncertainties were much smaller than log‐file uncertainties.^28^ Consequently, the position calibration of the setup might be improved in the future by taking the planned positions of the calibration irradiation as a reference instead of the log‐files positions. In this case, due to SCICOPRO spatial resolution, the pulses corresponding to a given PB position could be grouped easily without use of the log‐files identification. Nevertheless, a study of this calibration process should be performed to evaluate the gain (or not) on uncertainties, given the summation of pulse images and maybe the summation of delivery uncertainties.

Contrary to the position and energy measurements, this pulse‐by‐pulse study contributed to show that, the intensity uncertainties depend on the number of pulses per PB, with relative uncertainties ranging between 1.79% and 8.87% (average value + standard deviation) for the irradiations at 0.02 and 1  MU of section 3.1 respectively. Indeed, whatever the planned PB intensity (between 0.02 to about 4 MU in this study), it was divided into pulses whose intensities ranged between about 1.5 and 150 × 10^−3^ MU by the Proteus®One BGA delivery strategy. The drawback of this was thus the summation of systematic errors, in particular for the largest numbers of pulses per PB. On the other hand, this was also an advantage as the reduced range of intensities measured by the camera facilitated the optimization of the system sensitivity (i.e., maximizing optical aperture for low intensity signal without saturating for high intensity signals). As for the positions and energy measurements, PBs intensities may be calibrated on planned data, which could avoid the summation of systematic errors. Nevertheless, it could be more difficult to implement principally because of the spatial dependence of the measured signal for a given PB intensity due to many optical phenomena such as the optical vignetting, the diffusion, the perspective.^29^ The calibration irradiation presented in the Section 2.4 relied only on two intensities in this work (0.1 and 1 MU). It may be necessary to repeat it at multiple intensities for a calibration at the PB level, since the linearity of the camera response (which will remain correlated to the pulse acquisition anyway) will no longer be taken into account and corrected by the calibration and should be verified.

For the QA of delivered PB both calibration approach could be considered, the choice being driven by performance consideration. On the contrary, when pulse‐by‐pulse QA is of interest (for dose rate evaluation for example), it seems that a calibration of intensity relying on log‐files data would be the best approach, even if the use of a low thickness ionization chamber could also be considered.

Finally, SCICOPRO was efficient to detect treatment errors external to the Proteus®ONE system, simulated by small misalignments of the treatment table in this study. Other errors external to the Proteus®ONE system could occur, such as a deviation between the imaging and the irradiation isocenters for example.^30^ The detection of such external errors is not possible with the sole analysis of log‐files data and necessarily requires an independent detection system. SCICOPRO also advantageously allows identifying from which PB discrepancy comes from. Measured PB (or pulse) deviations could then be translated into dose discrepancies through Monte Carlo simulations of the dose deposition either in a homogeneous phantom for experimental verification of the simulated dose distribution or in the heterogeneous patient volume.

For homogeneous phantom dosimetry, the use of SCICOPRO could also be very advantageous as it has the potential to provide experimental dose distributions from the scintillation distributions.^17^, ^19^, ^20^, ^21^, ^31^ It would allow the complete verification of delivered PBs characteristics, as well as the comparison between simulated and measured dose distributions in a single irradiation. The implementation of optical artifacts and scintillation quenching corrections^19^, ^29^, ^32^, ^33^ is currently under progress to go toward 3‐D dosimetry. This could provide a complete verification tool which could even integrate dose rate information from pulse intensity data. This PSQA, which could be done preliminary to patient treatment, would be of great interest and complementary to PSQA performed after the treatment from recorded log‐files data.^23^, ^24^, ^25^

The setup presented in this paper was designed to verify small size irradiation fields, but a larger prototype based on a scintillator of 25 × 25 × 25 cm^3^ is also under investigation. Although optical biases of greater magnitude are expected with a larger scintillator, this work has shown that these spatially dependent artefacts can be corrected by a calibration based on PBs uniformly distributed throughout the scintillator volume. Although compromises between field of view, depth of focus and spatial resolution may be necessary, a larger system would be advantageous the verification of larger irradiation fields.

## 5 CONCLUSION AND PERSPECTIVE

In this work we developed a scintillation system based on a 10 × 10 × 10 cm^3^ and a fast camera recording two views (direct and through a mirror) of the scintillation distribution generated by the proton beams. The system was able to work on a pulse‐by‐pulse basis in PBS proton therapy thanks to a high acquisition rate (>1 kHz) and a very high sensitivity allowing the detection of pulse intensities as low as 2 × 10^−3^ MU, which corresponds to approximately 10^−4^ Gy at the Bragg peak position. The analysis of the scintillation distributions provided the PBs characteristics (position, energy, intensity in terms of delivered MU) with uncertainties (average value + standard deviation) smaller than 580 µm for the position, 180 keV for the energy and 3% for the intensity on patients’ treatment plans. SCICOPRO thus proved to be a fast and accurate tool for pulse or PB verifications in PSQA.

## CONFLICT OF INTEREST STATEMENT

The authors declare no conflicts of interest.
